# REG3A: A Potential Fecal Biomarker for Ulcerative Colitis

**DOI:** 10.1155/mi/9316792

**Published:** 2026-06-25

**Authors:** Xinrui Lv, Yaxin Qi, Jia Guo, Ling Ba, Sipu Wang, Hailong Cao, Xin Xu

**Affiliations:** ^1^ Department of Gastroenterology and Hepatology, Tianjin Medical University General Hospital, Tianjin, China, tjmugh.com.cn

**Keywords:** fecal calprotectin, regenerating islet-derived protein 3-alpha, ulcerative colitis

## Abstract

Regenerating islet‐derived protein 3‐alpha (REG3A), a member of the regenerating islet‐derived protein (Reg) family, belongs to the C‐type lectin class of antimicrobial peptides (AMPs). It has been recognized as a critical component of intestinal mucosal barrier damage and repair. In a prospective cohort (82 ulcerative colitis [UC] patients, 20 controls), UC patients were stratified by clinical partial Mayo Score (pMS) and endoscopic activity. Fecal REG3A levels were measured across distinct patient cohorts, and diagnostic performance was evaluated and compared against fecal calprotectin (FC) and a combined model using receiver operating characteristic (ROC) analysis. REG3A expression was also evaluated in intestinal biopsies via immunohistochemistry. Fecal REG3A levels increased stepwise with UC clinical severity (*p*  < 0.001), peaking in the severe activity group. A combined model of REG3A and FC outperformed either biomarker alone in differentiating disease activity, achieving the highest area under the curves (AUCs) for distinguishing remission from mild activity and mild from moderate activity. Immunohistochemistry confirmed REG3A expression in active UC colonic tissues. Our results indicate that fecal REG3A serves as a promising biomarker for assessing disease activity in UC. Its combination with FC enhances the accuracy of predicting clinical severity in UC.

## 1. Introduction

Ulcerative colitis (UC) is a chronic and progressive intestinal disorder characterized by continuous inflammatory changes extending proximally from the distal colon [1], predominantly affecting the mucosal and submucosal layers of the colon. The classic clinical presentation includes diarrhea, abdominal pain, and hematochezia [2].

Although the etiology of UC remains incompletely understood, growing evidence indicates that impaired intestinal mucosal barrier function represents a pivotal event in its pathogenesis [3]. Some experts have even proposed conceptualizing UC as an “intestinal barrier dysfunction syndrome” [4]. Compromised mucosal barrier integrity leads to increased intestinal permeability, bacterial and endotoxin translocation, and immune dysregulation mediated by inflammatory cell infiltration [5].

Current management strategies for UC have evolved from merely controlling symptoms toward achieving clinical remission (characterized by the absence of rectal bleeding and normalization of bowel frequency) and endoscopic remission (defined by mucosal healing), with the ultimate goal of halting disease progression and improving long‐term outcomes. An accurate assessment of disease activity is therefore crucial in UC [6–8]. The endoscopic Mayo score’s limitations for dynamic monitoring necessitate reliable, noninvasive biomarkers to reflect intestinal inflammation. Such biomarkers are urgently needed as practical, real‐time surrogates for endoscopy in inflammatory bowel disease (IBD) management [9]. Fecal calprotectin (FC) demonstrates high sensitivity for detecting mucosal inflammation, monitoring treatment response, and predicting clinical relapse [10].

The impairment of the intestinal mucosal barrier in UC triggers a stress response in Paneth cells. These specialized secretory cells respond by releasing an array of antimicrobial peptides (AMPs) [11]. Upon mucosal damage, regenerating islet‐derived protein 3‐alpha (REG3A) functions as a compensatory AMP and is released into the circulation, serving as a practical serum biomarker for intestinal barrier injury [12]. Serum REG3A was elevated in mucosal enteropathies versus IBS, with 77% of UC patients exceeding the IBS median. Correspondingly, mucosal gene expression identified REG3A as a disease‐relevant marker, demonstrating significant upregulation in IBD colonic mucosa but not in healthy controls, highlighting its link to gut inflammation [13]. Moreover, our preliminary studies demonstrated markedly upregulated intestinal REG3A protein expression in a DSS‐induced colitis animal model [14].

UC is a chronic IBD characterized by relapsing and remitting intestinal inflammation [15]. Accurate assessment of mucosal healing has emerged as a key therapeutic endpoint in UC management. Although elevated levels of both FC and fecal REG3A demonstrate sensitivity in detecting mucosal inflammation [16], the specificity of their combined prediction for confirming mucosal healing remains unvalidated. Consequently, there is a pressing need to identify novel biomarkers that can more directly reflect intestinal mucosal barrier status and inflammatory activity, thereby addressing a critical gap in current UC management strategies [17].

This prospective study tested the hypothesis that combining fecal REG3A with FC improves noninvasive prediction of mucosal healing in UC. Using binary logistic regression, we developed a clinical prediction model.

The study aimed to evaluate the utility of fecal REG3A, alone and combined with FC, for predicting disease activity and mucosal healing.

## 2. Methods

### 2.1. Patients and Study Design

The UC cohort consisted of consecutive patients in either active or remission stages, recruited during routine outpatient visits or hospitalization for disease flares, with fecal samples obtained accordingly. Patients with UC were assessed using the full Mayo scoring system, which evaluates disease activity based on clinical manifestations, imaging findings, endoscopic results, and histological examination. Disease activity was also assessed using the partial Mayo Score (pMS). Exclusion criteria for UC patients included a history of colorectal surgery; pregnancy or lactation; colitis associated with cytomegalovirus or *Clostridium difficile* infection; and severe cardiovascular, hepatic, renal, or respiratory diseases in order to avoid overestimation of the overall assessment component of the Mayo score. Control subjects were recruited from individuals undergoing routine health checkups. Inclusion criteria for controls were as follows: (1) no history of IBD or other chronic gastrointestinal disorders; (2) no gastrointestinal symptoms in the past 3 months; and (3) normal colonoscopy findings if performed. Exclusion criteria for controls were as follows: (1) pregnancy; (2) active infection; and (3) malignancy. All participants provided written informed consent [18, 19]. This study received ethical approval from the Clinical Research Ethics Committee of Tianjin Medical University General Hospital (Approval Number: IRB2022‐KY‐412). Mayo score definitions were as follows: clinical remission: a total score of ≤2 points, all subscores ≤1, and an endoscopic subscore of 0 or 1; mild active phase: 3–5 points; moderate active phase: 6–10 points; and severe active phase: 11–12 points. Disease location and clinical phenotype were classified according to the Montreal classification [20]. Endoscopic activity in UC patients was assessed using the UC Endoscopic Index of Severity (UCEIS) [21] and the Mayo endoscopic subscore [18, 19] (remission defined as ≤1).

### 2.2. Fecal Sample Collection and Processing

Fecal samples were collected from each participant using a standard sterile container. Patients were instructed to avoid mixing urine or water with the stool sample. Samples were transported to the laboratory immediately after collection (within 2 h) under cold chain conditions (4°C). Upon arrival, ~100–200 mg of feces was aliquoted and stored at −80°C until analysis. The storage duration ranged from 1 to 6 months. All samples were limited to a single freeze–thaw cycle to prevent protein degradation.

### 2.3. Enzyme‐Linked Immunosorbent Assay (ELISA) for Human REG3A

Stool REG3A concentrations were measured using a commercial ELISA kit (SenBeiJia Biological Technology Co., Ltd., Nanjing, China). The assay has a detection range of 100–4000 pg/mL, with intra‐assay and interassay coefficients of variation <9% and <11%, respectively, and a linear correlation coefficient >0.990. Fecal samples were thawed, homogenized in PBS, and centrifuged at 2000–3000 rpm for 20 min at 4°C. The supernatant was assayed in duplicate following the manufacturer’s protocol, and concentrations were determined using a standard curve.

### 2.4. Clinical Laboratory Data

On the day of fecal collection, clinically relevant laboratory data—including FC, erythrocyte sedimentation rate (ESR), serum C‐reactive protein (CRP), and hemoglobin—were obtained and analyzed using standard hospital laboratory procedures.

### 2.5. Endoscopic Assessment

All UC patients underwent colonoscopy with the endoscopist being blinded to biomarker data. Endoscopic activity was graded using the Mayo endoscopic subscore (0: normal/inactive; 1: mild disease [erythema, decreased vascular pattern, mild friability]; 2: moderate disease [marked erythema, absent vascular pattern, friability, and erosions]; and 3: severe disease [spontaneous bleeding, ulceration]) by an IBD specialist (F.M.) based on reviewed reports and images. Endoscopic remission was defined as a Mayo score of 0 [19].

### 2.6. Immunohistochemical Assessment

Colon biopsies from IBD patients (remission, *n* = 1; active, *n* = 1) and healthy volunteers (*n* = 1) were processed routinely. IHC for REG3A was performed on formalin‐fixed paraffin‐embedded (FFPE) tissue using an automated BenchMark ULTRA system (Ventana) with a specific rabbit antihuman antibody (Affinity, DF6825; 1:100), followed by UltraView Universal DAB detection.

### 2.7. Statistical Analyses

Spearman’s correlation test was used to assess the associations between variables. Receiver operating characteristic (ROC) curves were generated to evaluate the diagnostic performance of REG3A, FC, and their combination. A binary logistic regression model was constructed to generate the combined prediction model (Combination), with disease activity as the dependent variable and REG3A and FC as independent variables using the enter method. The predicted probability from this model was used as the combination predictor. Area under the curve (AUC) values were compared using DeLong’s test, with statistical significance set at *p*  < 0.05. All analyses were performed using SPSS Statistics version 25.0 (IBM Corp.) and GraphPad Prism version 8.00 (GraphPad Software).

## 3. Results

### 3.1. Cohort Characterization and Disease Outcomes in Clinical Standard

**Figure 1:**
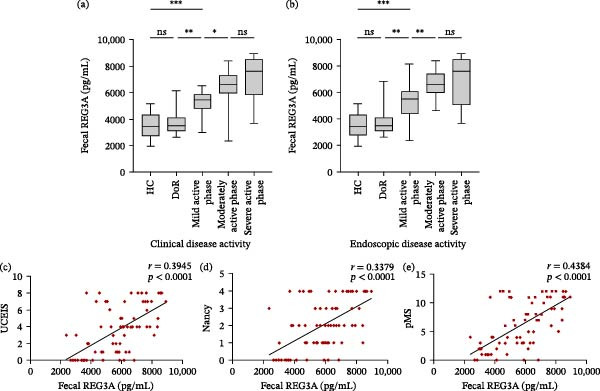
Comparison of fecal REG3A levels in healthy controls and patients with UC regarding (a) clinical remission and activity and (b) endoscopic remission and activity. Correlation curves between fecal REG3A levels and (c) UCEIS scores, (d) Nancy scores, and (e) pMS scores. REG3A, regenerating islet‐derived protein 3‐alpha.  ^∗^ *p*  < 0.05,  ^∗∗^ *p*  < 0.01,  ^∗∗∗^ *p*  < 0.001.

UC patients (*n* = 82) were stratified by pMS into clinical remission (*n* = 19, 23.17%), mild (*n* = 19, 23.17%), moderate (*n* = 22, 26.83%), and severe (*n* = 22, 26.83%) activity groups (Table 1). Fecal REG3A correlated positively with pMS (Figure 1e).

**Table 1:** Demographic and clinical staging characteristics of the study population.

Clinical activity	Remission	Mild active phase	Moderately active phase	Severe active phase	*p*
Patients	19	19	22	22	—
Age	58.84 ± 11.58	47.63 ± 18.91	57.10 ± 18.50	50.29 ± 17.16	0.0756
Male (%)	14 (73.68)	12 (57.89)	15 (68.18)	14 (63.64)	0.8877
Disease duration (year)	5.68 ± 6.66	4.63 ± 3.18	4.1 ± 2.99	4.9 ± 3.58	0.7591
ESR (mm/h)	16.11 ± 11.43	19.26 ± 11.51	22.38 ± 12.68	34.62 ± 18.65	0.0003
CRP (mg/L)	1.59 ± 2.02	2.72 ± 3.71	3.33 ± 2.83	4.03 ± 3.63	0.0533
FC (ng/mL)	200.69 ± 62.85	322.38 ± 113.52	571.47 ± 231.09	833.82 ± 369.28	<0.0001
pMS	1 ± 0.74	3.68 ± 0.73	7.73 ± 1.26	11.52 ± 0.5	<0.0001
Hemoglobin (g/L)	113.47 ± 22.57	117.32 ± 21.57	121.52 ± 21.49	106.19 ± 36.10	0.3276
Reg3A (pg/mL)	3774.24 ± 965.58	5296.79 ± 936.93	6515.41 ± 1327.93	7215.15 ± 1540.92	<0.0001
Montreal extent					<0.001
E1 (%)	12 (63.16)	7 (36.84)	3 (13.64)	2 (9.09)	—
E2 (%)	5 (26.32)	6 (31.57)	4 (18.18)	8 (36.36)	—
E3 (%)	2 (10.52)	6 (31.57)	15 (68.18)	12 (54.55)	—
Medication					0.0690
5‐ASA (%)	3 (15.79)	4 (21.05)	7 (31.82)	2 (9.09)	—
Thiopurin (%)	1 (5.26)	0	2 (9.09)	2 (9.09)	—
Infliximab (%)	1 (5.26)	1 (5.26)	0	1 (4.55)	—
Vedolizumab (%)	14 (73.68)	13 (68.42)	6 (27.27)	12 (54.55)	—
Ustekinumab (%)	0	1 (5.26)	0	0	—
Upadacitinib (%)	0	0	7 (31.82)	5 (22.73)	—

*Note:* E1—proctitis; E2—left‐sided colitis; E3—pancolitis.

Abbreviations: 5‐ASA, 5‐aminosalicylic acid; CRP, serum C‐reactive protein; ESR, erythrocyte sedimentation rate; FC, fecal calprotectin; pMS, partial Mayo Score; REG3A, Regenerating islet‐derived protein 3‐alpha.

The distribution of age, sex, and disease duration was similar across the different disease activity groups. ESR and FC increased stepwise with clinical severity (*p*  < 0.05), with the lowest levels in clinical remission and highest in severe activity. Disease extent (Montreal classification) was unbalanced across groups: proctitis (E1) was most prevalent in the remission group (63.16%), left‐sided colitis (E2) was more common in the moderate group (36.36%), and extensive colitis (E3) was predominant in the severe group (68.18%). Treatment regimens differed: 5‐aminosalicylic acid (5‐ASA) was common in moderate activity (31.82%) and glucocorticoids in moderate‐to‐severe activity (9.09%). Biologic use varied: infliximab in remission and mild activity (5.26%), vedolizumab in remission (73.68%), ustekinumab in mild activity (5.26%), and upadacitinib in moderate activity (31.82%). Key findings are summarized in Table 1.

Fecal REG3A levels were significantly elevated in active UC (mild: 5296.79 ± 936.93 pg/mL, moderate: 6515.41 ± 1327.93 pg/mL, severe: 7215.15 ± 1540.92 pg/mL) compared to remission (3774.24 ± 965.58 pg/mL) and healthy controls (3521.00 ± 870.91 pg/mL), showing a stepwise increase with disease severity (*p*  < 0.001) (Figure 1a, Table 1).

**Figure 2:**
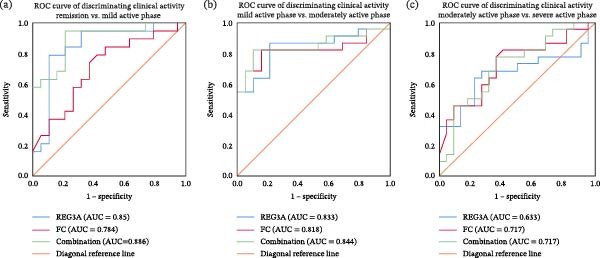
ROC analysis of fecal REG3A and FC, the combination model in predicting (a) clinical remission and mild clinical activity, (b) mild clinical activity and moderate clinical activity, (c) moderate clinical activity and severe clinical activity. The diagonal orange line indicates chance performance (AUC = 0.5). Pairwise AUC comparisons were performed using DeLong’s test, with detailed results presented in Table 2. Sample sizes: remission (*n* = 19), mild (*n* = 19), moderate (*n* = 22), severe (*n* = 22). AUC, area under the curve; REG3A, regenerating islet‐derived protein 3‐alpha.

To evaluate fecal REG3A, FC, and their combination model, ROC curve analysis was performed comparing patients in clinical remission to the mild active phase. The analysis demonstrated significant diagnostic utility for all three indicators (each *p*  < 0.001). The combination model achieved the highest AUC value (AUC = 0.886 [CI: 0.772–1.000], *p*  < 0.001), followed by REG3A (AUC = 0.85 [CI: 0.717–0.984], *p*  < 0.001), and FC (AUC = 0.784 [CI: 0.638–0.929], *p* = 0.003) (Figure 2a).

**Table 2:** DeLong test *p*‐values for pairwise comparisons of AUCs among FC, RRG3A, and the combined model.

Activity	Type comparison	Model	AUC (CI)	*p*‐Value (DeLong)
Clinical	Remission vs. mild	Combination vs. FC	0.102 (−0.023–0.228)	0.308
		Combination vs. REG3A	0.036 (−0.033–0.105)	0.109
		REG3A vs. FC	0.066 (−0.096–0.229)	0.424
	Mild vs. moderate	Combination vs. FC	0.026 (−0.011–0.064)	0.166
		Combination vs. REG3A	0.012 (−0.071–0.095)	0.777
		REG3A vs. FC	0.014 (−0.094–0.122)	0.795
	Moderate vs. severe	Combination vs. FC	−0.004 (−0.08–0.072)	0.915
		Combination vs. REG3A	0.050 (−0.132–0.231)	0.592
		REG3A vs. FC	−0.054 (−0.172–0.065)	0.374
Endoscopic	Remission vs. mild	Combination vs. FC	0.101 (−0.045–0.248)	0.175
		Combination vs. REG3A	0.018 (−0.024–0.061)	0.398
		REG3A vs. FC	0.083 (−0.084–0.25)	0.330
	Mild vs. moderate	Combination vs. FC	0.005 (−0.018–0.028)	0.688
		Combination vs. REG3A	0.055 (−0.024–0.134)	0.176
		REG3A vs. FC	−0.05 (−0.135–0.035)	0.247
	Moderate vs. severe	Combination vs. FC	0.008 (−0.089–0.104)	0.877
		Combination vs. REG3A	0.043 (−0.157–0.244)	0.672
		REG3A vs. FC	−0.036 (−0.157–0.086)	0.565

Abbreviations: FC, fecal calprotectin; REG3A, regenerating islet‐derived protein 3‐alpha.

ROC curve analysis was also performed comparing patients in the mild active phase to the moderate active phase. All three indicators demonstrated significant diagnostic utility. The combination model achieved the highest AUC value (AUC = 0.844 [CI: 0.715–0.974], *p*  < 0.001), followed by REG3A (AUC = 0.833 [CI: 0.702–0.963], *p*  < 0.001), and FC (AUC = 0.818 [CI: 0.677–0.960], *p* = 0.001) (Figure 2b).

For the comparison between moderate and severe UC activity, ROC analysis demonstrated significant diagnostic utility only for FC and the combination model. FC showed discriminatory power (AUC = 0.717 [CI: 0.563–0.871], *p* = 0.014), while REG3A alone did not reach statistical significance (AUC = 0.663 [CI: 0.494–0.833], *p* = 0.064). The combination model (AUC = 0.717 [CI: 0.559–0.867], *p* = 0.016) did not outperform FC alone (Figure 2c).

However, as shown in Table 2, DeLong’s test revealed that none of the pairwise AUC differences between the combination model and either single biomarker reached statistical significance (all *p*  > 0.05). Therefore, while the combination model showed numerically higher AUC values in certain comparisons, these advantages did not achieve statistical significance in our cohort.

### 3.2. Cohort Characterization and Disease Outcomes in Endoscopic Standard

UC patients (*n* = 82) were stratified by Mayo endoscopic score into endoscopic remission (*n* = 19, 23.17%), mild (*n* = 20, 24.39%), moderate (*n* = 21, 25.61%), and severe (*n* = 22, 26.83%) activity groups (Table 3). Fecal REG3A correlated positively with UCEIS (Figure 1c) and the histological Nancy Index (Figure 1d).

**Table 3:** Demographic and endoscopic staging characteristics of the study population.

Endoscopic activity	Remission	Mild active phase	Moderately active phase	Severe active phase	*p*
Patients	19	20	21	22	—
Age	54.6 ± 13.9	52.3 ± 16.9	54.7 ± 19.9	50.9 ± 17.2	0.6406
Male (%)	15 (78.95)	13 (65.0)	14 (66.47)	13 (59.09)	0.5959
Disease duration (year)	5.37 ± 6.73	4.55 ± 3.19	4.71 ± 3.12	5.27 ± 4.19	0.9243
ESR (mm/h)	15.95 ± 11.44	17.5 ± 11.25	25.43 ± 14.88	37.10 ± 27.19	0.0009
CRP (mg/L)	1.54 ± 2.02	2.91 ± 4.09	3.34 ± 2.53	4.69 ± 5.55	0.0895
FC (ng/mL)	214.51 ± 102.56	327.61 ± 167.1	603.11 ± 207.61	789.55 ± 391.16	<0.0001
Hemoglobin (g/L)	113.05 ± 22.34	119.55 ± 19.6	120.95 ± 23.93	104.41 ± 35.23	0.1691
REG3A (pg/mL)	3888.57 ± 1172.66	5285.79 ± 1305.89	6622.91 ± 985.68	6983.42 ± 1661.97	<0.0001
Montreal extent					<0.001
E1 (%)	12 (63.16)	8 (40.0)	2 (9.52)	2 (9.09)	—
E2 (%)	5 (26.32)	7 (35.0)	3 (14.29)	8 (36.36)	—
E3 (%)	2 (10.52)	5 (25.0)	16 (76.19)	12 (54.55)	—
Medication					0.0302
5‐ASA (%)	3 (15.79)	5 (25.0)	7 (33.33)	2 (9.09)	—
Thiopurin (%)	1 (5.26)	0	2 (9.52)	1 (4.55)	—
Infliximab (%)	1 (5.26)	1 (5.0)	0	1 (4.55)	—
Vedolizumab (%)	14 (73.68)	14 (70.0)	4 (19.05)	13 (59.09)	—
Ustekinumab (%)	0	0	1 (4.76)	0	—
Upadacitinib (%)	0	0	7 (33.33)	5 (22.73)	—

*Note:* E1—proctitis; E2—left‐sided colitis; E3—pancolitis.

Abbreviations: 5‐ASA, 5‐aminosalicylic acid; CRP, serum C‐reactive protein; ESR, erythrocyte sedimentation rate; FC, fecal calprotectin; REG3A, Regenerating islet‐derived protein 3‐alpha.

Patient demographics, including age, sex, and disease duration, were similar across disease activity groups. ESR FC and REG3A increased stepwise with clinical severity (*p*  < 0.05), with the lowest levels in endoscopic remission and highest in endoscopic severe activity. Disease extent (Montreal classification) was unbalanced across groups: proctitis (E1) was most prevalent in the remission group (63.16%), left‐sided colitis (E2) was more common in the mild (35.0%) and severe (36.36%) groups, and extensive colitis (E3) was predominant in the moderate (76.19%) and severe (54.55%) groups. Treatment regimens differed: 5‐aminosalicylic acid (5‐ASA) was common in moderate activity (33.33%); corticosteroids in moderate activity (9.09%). Biologic therapies used varied: infliximab in remission (5.26%), vedolizumab in endoscopic remission (73.68%), and ustekinumab and upadacitinib both in moderate activity (4.76% and 33.33%). Key findings are summarized in Table 3.

Fecal REG3A levels were significantly elevated in active UC (mild: 5285.79 ± 1305.89, moderate: 6622.91 ± 985.68, severe: 6983.42 ± 1661.97 pg/mL) compared to remission (3888.57 ± 1172.66 pg/mL) and healthy controls (3521.00 ± 870.91 pg/mL), showing a stepwise increase with disease severity (*p*  < 0.001) (Figure 1b, Table 3).

**Figure 3:**
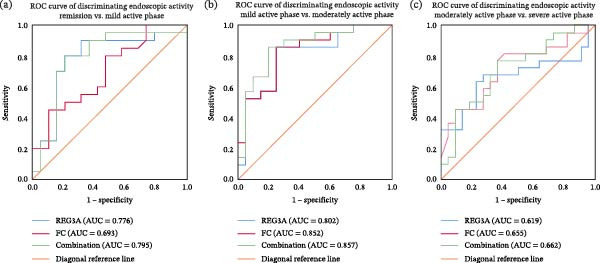
ROC analysis of fecal REG3A and FC, the combination model in predicting (a) endoscopic remission and mild endoscopic activity, (b) mild endoscopic activity and moderately endoscopic activity, and (c) moderately endoscopic activity and severe endoscopic activity. The diagonal orange line indicates chance performance (AUC = 0.5). Pairwise AUC comparisons were performed using DeLong’s test, with detailed results presented in Table 2. Sample sizes: remission (*n* = 19), mild (*n* = 20), moderate (*n* = 21), severe (*n* = 22). AUC, area under the curve; REG3A, regenerating islet‐derived protein 3‐alpha.

ROC curve analysis was performed to compare patients in endoscopic remission to the mild active phase. The analysis demonstrated significant diagnostic utility for fecal REG3A, FC, and their combination models. The combination model achieved the highest AUC value [AUC = 0.795 (CI: 0.642–0.947), *p* = 0.002], followed by REG3A [AUC = 0.776 (CI: 0.616–0.984), *p* = 0.003] and FC [AUC = 0.693 (CI: 0.528–0.859), *p* = 0.039] (Figure 3a).

ROC curve analysis was also performed comparing patients in the mild active phase to the moderate active phase. All three indicators demonstrated significant diagnostic utility. The combination model achieved the highest AUC value [AUC = 0.857 (CI: 0.738–0.976), *p*  < 0.001], followed by REG3A [AUC = 0.802 (CI: 0.664–0.941), *p*  < 0.001] and FC [AUC = 0.852 (CI: 0.734–0.971), *p* = 0.001] (Figure 3b).

For distinguishing between moderate and severe active phases, neither REG3A, FC, nor the combination model demonstrated statistically significant diagnostic performance (all *p*  > 0.05). However, a nonsignificant trend was observed, with the combination model showing the highest AUC value [AUC = 0.662 (CI: 0.499–0.826), *p* = 0.068], followed by FC [AUC = 0.665 (CI: 0.488–0.822), *p* = 0.082] and REG3A [AUC = 0.619 (CI: 0.440–0.798), *p* = 0.181] (Figure 3c).

Thus, although the combination model showed numerically higher AUC values in these comparisons, none of these advantages reached statistical significance according to DeLong’s test (all *p*  > 0.05; Table 2).

### 3.3. REG3A Immunohistochemical Evaluation

**Figure 4:**
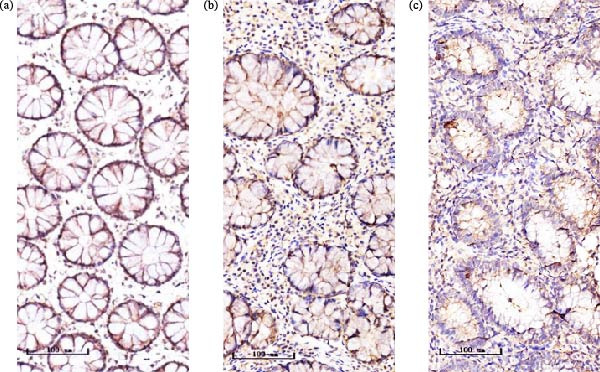
Immunohistochemical expression of REG3A in (a) healthy control, (b) UC in both clinical and endoscopic remission, and (c) UC with clinical and endoscopic activity. Scale bar = 100 μm. REG3A, regenerating islet‐derived protein 3‐alpha; UC, ulcerative colitis.

Examples of REG3A immunohistochemical localization in healthy volunteers and UC patients are shown. Normal colonic mucosa (Figure 4a) exhibited no epithelial REG3A expression. Remission UC patients (Figure 4b) showed REG3A at the apical epithelial surface, while active UC patients (Figure 4c) demonstrated a similar apical expression.

## 4. Discussion

REG3A, a member of the regenerating islet‐derived protein (Reg) family [22], belongs to the C‐type lectin class of AMPs [23]. The mouse Reg III gene family primarily comprises three members: RegIIIα, RegIIIβ, and RegIIIγ, whereas humans possess only two homologs: RegIIIα and RegIIIγ. Human RegIIIα, (also known as hepatocarcinoma–intestine–pancreas/pancreatic‐associated protein [HIP/PAP]), shares 67% homology with murine RegIIIγ and is expressed in both normal small intestine and inflamed colonic tissues [24]. Intestinal RegIIIγ/HIP/PAP, predominantly synthesized and secreted by small intestinal Paneth cells and enterocytes, exerts direct antibacterial effects through specific binding to bacterial peptidoglycan [25]. Its upregulation in active UC mucosa likely reflects a concerted effort to restore epithelial homeostasis and contain translocating microbes.

The expression of REG3A (HIP/PAP) is dependent on gut microbiota colonization and is mediated through microbial‐host interactions in the intestinal mucosa. The gut microbiota, through antigen presentation, activates intestinal epithelial signaling pathways that drive REG3A synthesis. During impaired intestinal barrier function, microbial components engage pattern recognition receptors to trigger the MyD88‐dependent signaling pathway within enterocytes, directly upregulating REG3A production [26]. Furthermore, microbiota‐stimulated Th17 cells and group 3 innate lymphoid cells (ILC3s) secrete IL‐22, which activates the STAT3 pathway in intestinal epithelial cells, inducing the synthesis and secretion of RegIII&gamma [27]. Consequently, fecal REG3A levels provide a direct measure of host–microbe interaction intensity at the mucosal interface. Our study demonstrates that fecal REG3A serves as a novel and promising noninvasive biomarker for discerning disease activity in UC. The progressive elevation of REG3A levels across escalating severities of endoscopic and clinical activity, coupled with its robust performance in ROC analyses, underscores its potential clinical utility.

A critical question is how REG3A compares to the current gold standard, FC. Both biomarkers performed well, yet key distinctions may define their respective niches. While FC is a neutrophil‐derived protein that quantifies phagocyte infiltration [28], REG3A originates directly from the Paneth cell [23]. This suggests that REG3A might be a more proximate marker of epithelial stress and repair mechanisms. Future studies with larger cohorts are warranted to determine if REG3A offers superior sensitivity or a faster normalization upon therapeutic intervention.

Therefore, in contrast to FC, which reflects neutrophil infiltration, the elevated levels of REG3A—directly secreted by Paneth cells—represent not merely an inflammatory consequence but rather an active host defense and repair mechanism [29]. Consequently, REG3A emerges as a biomarker that directly mirrors the intestinal epithelial cell status and barrier function.

As an AMP, REG3A has been investigated in the context of UC and IBD, with existing studies primarily focusing on its mechanistic role in protecting the intestinal barrier [30]. As a biomarker, REG3A expression has been shown to be elevated in the inflamed colonic mucosa of IBD patients, and Reg3a levels can distinguish mucosal enteropathy from irritable bowel syndrome with 90% sensitivity and 96% specificity, supporting its utility as a marker of intestinal barrier dysfunction [12]. However, current evidence does not address the use of REG3A as a biomarker for assessing disease activity in UC; most published studies have only compared UC patients with healthy controls. In contrast, our study not only validates REG3A as a biomarker for evaluating disease activity in UC but also demonstrates that the combination of REG3A with FC shows promising diagnostic performance, warranting further validation in larger cohorts.

Limitations include the unestablished specificity of fecal REG3A for UC versus other inflammatory conditions, its potential role as a general marker of barrier injury, and the homogeneous, single‐center cohort, which may limit generalizability.

The lack of statistical significance despite numerically higher AUC may be attributed to the limited sample size, which may have underpowered the DeLong test to detect modest differences. Alternatively, the additional predictive contribution of REG3A beyond FC alone may be modest in this cohort. Larger prospective studies are warranted to validate the added value of the combination model. Additionally, due to the limited sample size of patients with proctitis (E1 disease), we did not perform subgroup analyses based on disease extent, and therefore, the potential impact of disease extent on diagnostic performance could not be adequately addressed.

It should be noted that our immunohistochemical findings are based on a limited number of samples and are preliminary in nature. These images are intended solely to demonstrate the localization of REG3A expression and are not intended for quantitative evaluation. No firm biological conclusions can be drawn from these data alone. Future studies with larger sample sizes and quantitative analysis are required to confirm the tissue expression pattern of REG3A.

Furthermore, clinical translation requires assay standardization and validated cutoffs.

Fecal REG3A represents a compelling biomarker for UC activity, providing an epithelial perspective complementary to FC. As a potential adjunct to current monitoring [31], it may refine response prediction and patient stratification, though prospective studies are needed to define its role in personalized management.

## 5. Conclusions

Our results indicate that fecal REG3A serves as a promising biomarker for assessing the disease activity in UC. Its combination with FC enhances the accuracy of predicting clinical severity in UC.

## Author Contributions

**Xinrui Lv**: conceptualization, methodology, investigation, writing – original draft. **Yaxin Qi**: conceptualization, writing – review and editing. **Jia Guo**: data curation, writing – review and editing. **Ling Ba**: software, writing – review and editing. **Sipu Wang**: software, writing – review and editing. **Hailong Cao**: conceptualization, resources, project administration, funding acquisition. **Xin Xu**: conceptualization, methodology, resources, writing – review and editing, supervision, project administration, funding acquisition.

## Funding

This research was supported by the Qingfeng Scientific Research Fund of the China Crohn’s & Colitis Foundation (CCCF) (Grant CCCF‐QF‐2022C09‐24).

## Disclosure

All the authors identified and approved the article.

## Conflicts of Interest

The authors declare no conflicts of interest.

## Data Availability

The data will be made available upon request.
